# Adolescents’ Concerns, Routines, Peer Activities, Frustration, and Optimism in the Time of COVID-19 Confinement in Spain

**DOI:** 10.3390/jcm10040798

**Published:** 2021-02-16

**Authors:** Noelia Muñoz-Fernández, Ana Rodríguez-Meirinhos

**Affiliations:** 1 Department of Psychology, Universidad Loyola Andalucía, 41704 Seville, Spain; nmunoz@uloyola.es; 2 Department of Communication and Education, Universidad Loyola Andalucía, 41704 Seville, Spain

**Keywords:** COVID-19, adolescents, concerns, activities, frustration

## Abstract

The global outbreak of COVID-19 has brought changes in adolescents’ daily routines, restrictions to in-person interactions, and serious concerns about the situation. The purpose of this study was to explore COVID-19-related concerns, daily routines, and online peer activities during the confinement period according to sex and age groups. Additionally, the relationship of these factors and optimism along with adolescents’ frustration was examined. Participants included 1246 Spanish students aged 16–25 years old (M = 19.57; SD = 2.53; 70.8% girls). The results indicated that the top concern was their studies. COVID-19-related concerns, daily routines, and online peer activities varied by sex and age. Findings also revealed moderate to high levels of frustration, which were associated with adolescents’ main concerns, online peer activities, maintaining routines, and optimism. The results are discussed in light of their implications in designing support programs and resources to reduce the psychological impact of COVID-19 on adolescent mental health.

## 1. Introduction

The novel coronavirus disease (COVID-19) has become a major public health concern and was declared a pandemic by the World Health Organization in March 2020. Since the first cases were confirmed, the epidemic has rapidly spread worldwide. To control the outbreak, the Spanish government, similar to the governments of other countries all over the world, ordered a nationwide restrictive confinement for three months, starting from mid-March. During this period, a high restriction of mobility was imposed. Citizens were not allowed to leave their house except for essential reasons (e.g., buying supplies in the supermarket or pharmacy, taking care of vulnerable people, or going to work if teleworking was not possible). Schools were closed and other educational, social, cultural, artistic, sporting, or similar activities were canceled.

Although adolescents and young people are at lower risk of critical COVID-19 symptoms [1], strict confinement orders entailed important changes in daily routines and social interactions. With schools closed, youths had to adapt quickly to new remote learning environments, while uncertainties about their studies and their near academic future emerged [2]. These difficulties have been particularly marked among higher education students, who were preparing for university entrance exams, taking semester assessments, or doing practical training that could not adequately be replaced by online instruction [3]. Other extracurricular (e.g., educational and sports) and out-of-home leisure activities (e.g., hanging out with friends and dating), which provide valuable resources for socialization [4] were canceled, leaving adolescents with limited opportunities for face-to-face social contact. Although online social networks and instant messaging apps may have compensated for these shortages [5], there is emerging evidence that COVID-19 confinement has increased the risk of social isolation and loneliness among youths [6]. However, changes in interpersonal relationships go beyond friendships, also affecting family interactions. Family dynamics have had to change according to the stay-at-home mandates, forcing families to spend all their time together [7]. As a consequence, adolescents may have experienced restrictions in their personal space, while parents have faced an increase in daily stressors (including demands of caregiving and parenting, teleworking, home-schooling, threat of contagion, or financial insecurity, among others).

Exposure to COVID-19 challenges is having a substantial cost on psychological wellbeing [8,9]. To date, a growing number of studies among children and youths have reported high rates of anxiety and stress, along with difficulties with concentrating and worrying [10,11,12]. Although research is still progressing, it seems that from the very beginning, concerns about the consequences of COVID-19 have been particularly salient among young people. In early reports at the first stage of the outbreak in China, college students acknowledged being fearful of what was happening [13]. As this epidemic spread worldwide, this finding has been confirmed by more recent studies with adolescents and university students. These studies have found moderate levels of concern about COVID-19 [14], which were significantly related with increased anxiety and depression [15,16,17,18,19,20]. However, these worries may not be experienced by all young people in the same way. Indeed, studies indicate that levels of anxiety and fear about COVID-19 increase with age [14] and are higher among females [12,17,18,21]. Given these findings, research aimed at identifying the particular concerns that young people are experiencing would help to define better how they are coping with this crisis. To date, research has focused more on analyzing the overall levels of fear of COVID-19, e.g., [14,17,22], rather than describing adolescents’ reasons for these concerns. Among the few available sources of evidence, studies indicate that the main worries of adolescents revolve around the issues that currently cause more uncertainty: the health of those more vulnerable to COVID-19 and the economic situation [23,24]. Although less studied, school-related concerns may have also become magnified [12,25], especially when considering that the closure of schools during the pandemic is a global issue [2] and that education is of central importance for adolescents’ future.

Another important, yet less studied, consequence of the confinement may be frustration [26]. As shown by previous research, feelings of frustration emerge in situations in which people feel pressured to comply with rules which are perceived as a threat to their freedom [27]. Although adolescents may have different motivations to adhere to imposed measures, the confinement situation has certainly involved a number of restrictions on individual choice and decision-making (i.e., limitations on non-essential movements, prohibition of gathering with friends, and the obligation of wearing a face mask), which may have resulted in elevated levels of frustration. However, in the context of this pandemic, individual experiences of feeling thwarted may have been not only a common but a harmful consequence of the lockdown. Accordingly, ample research has well established that the psychological costs of frustration are related to higher levels of stress, depression, or anxiety, among others (see [28] for a review).

While there is no doubt that COVID-19 is having negative consequences in different areas of young people’s lives, research investigating the factors that help adolescents handle this stressful experience is very valuable [29]. Over the last months, social and health agencies have offered guidelines to support adolescents. However, empirical evidence on the factors that mitigate the risk of psychological distress is still lacking. Among these, experts have outlined the importance of establishing stay-at-home routines and doing a variety of activities (e.g., school-work, hobbies, and exercising) to reduce the psychological stress of the confinement [30], yet few studies have examined the role that these activities play in adolescent psychological functioning. On an interpersonal level, interactions with peers may also have an important influence on the way that adolescents experience this health crisis. In adolescence, peer relationships become especially salient as they contribute not only to satisfying the needs of intimacy and companionship but also to navigate the challenges of this developmental stage [31]. During the lockdown, youths stayed connected with their friends and classmates although face-to-face interactions moved to an online setting [32]. Technology and social media may have indeed helped to compensate for the lack of in-person interactions. Nonetheless, little is known about the specific online activities that youths have been engaging in with their peers, and the role of these activities in adolescent psychological outcomes during the lockdown. Finally, some authors have drawn attention to optimism as a factor that may favor better adaptive outcomes under challenging situations [31]. As such, recent studies demonstrated that keeping a more optimistic view of the situation was related to lower rates of anxiety and depressive symptoms during the COVID-19 pandemic [32,33].

The present study focused on the psychological impact of the COVID-19 lockdown in order to offer insights into the factors related to adolescents’ feelings of frustration. Specifically, the first aim was to describe adolescents’ main concerns about the impact of COVID-19. It was expected that school-related concerns [12,25], concerns about the health of those more vulnerable to COVID-19, and concerns about the economic situation [23,24] would be the most salient worries among young people. In addition, it was hypothesized that COVID-19-related concerns would be higher among females [12,17,18,21] and participants in higher age groups [14]. Second, this study explored the activities that, on a daily basis or with their peers, adolescents have been engaging in during the COVID-19 lockdown. Participants were expected to engage in several stay-at-home routines [30]. Besides, considering that social media by its nature may compensate for a lack of face-to-face social interactions [32], we hypothesized that youths may have been using technology to feel supported, loved, or cared for by their friends during the confinement period. Third, given that frustration experiences have significant costs on wellbeing and diminished functioning, this study also examined adolescents’ feelings of frustration during the confinement period and their link with COVID-19-related concerns, daily routines, online activities with peers, and optimism. Additionally, sex and age differences were examined. Adolescents were expected to display moderate to high levels of frustration [26]. As literature addressing the role of adolescents’ concerns and routines on frustration is still scant, the association between these variables was addressed in an exploratory fashion.

## 2. Materials and Methods

### 2.1. Participants and Procedure

The study sample consisted of 1246 students (70.8% girls) from Spain. The participant age range was 16–25 years (M = 19.57; SD = 2.53). Following Steinberg [34], participants in the 16 to 18 age group were considered middle adolescents, meanwhile, participants in the 19 to 25 age group were considered late adolescents. In this study, 42.3% (*n* = 527) were middle adolescents and 57.7% (*n* = 719) were late adolescents. The distribution of participants according to sex was similar between the younger (16–18 years old; 70.4% girls) and the older (19–25 years old; 71.07% girls) age groups, χ2(1) = 0.07, *p* > 0.05. In terms of geographic distribution, most participants (88.9%) came from the southern area of Spain (Andalucía), although students from all other regions of Spain were also represented in the sample. All respondents were students enrolled in compulsory secondary education or professional training (17.9%), post-secondary education (24%), or university (58.1%). During the confinement, participants were living in two-parent families (86.4%), single-parent families (7%), with other relatives (1.5%), with roommates and/or their partners (4.8%), or alone (0.3%).

Among the study sample, few participants (0.1%) and their relatives (3.1%) were diagnosed with COVID-19. Additionally, although testing for COVID-19 was not performed, other participants also indicated that they (7.1%) or their relatives (8.2%) had COVID-19-like symptoms. In terms of the socio-economic impact of the health crisis, more than half of the sample (52.4%) reported a significant reduction in their family’s income as a result of COVID-19.

Data were collected through an online survey using the Qualtrics software platform during the fifth–sixth week of the state of emergency in Spain (from 17 April to 1 May 2020). Participants were recruited through snowball sampling. The authors first distributed the online questionnaire through colleagues and potentially eligible participants who met the inclusion criteria: students between 16–25 years old, of Spanish nationality, and who were living in Spain. Then, initial participants were asked to send the questionnaire to other potential informants that met the inclusion criteria. The survey link was also shared through social media (Facebook, Instagram, Twitter, and WhatsApp), in the newsletter of the authors’ university, and with school institutions and local youth organizations. Of the 1582 students who actively consented to participate, 336 respondents were excluded from the study sample. Reasons for exclusion included the following: participants did not meet the inclusion criteria; the questionnaire was blank, or respondents only answered demographic questions; and the time spent to complete the questionnaire was less than 7 min, which is significantly faster (10th percentile) than the average (11 min).

The approval to conduct this study was obtained from the Ethics Committee of the Universidad Loyola Andalucía. Participation was voluntary and anonymity was guaranteed. Active informed consent was obtained prior to participation.

### 2.2. Measures

COVID-19-related concerns: An ad hoc five-item questionnaire was used to measure concerns about the impact of COVID-19 during the confinement. With the question “how much have you been worrying about…?” participants indicated their concerns related to: their own health (i.e., “getting ill with COVID-19”); the health of others (i.e., “my relatives getting ill with COVID-19”); their family financial strain, currently (i.e., “your family financial situation in this moment”) and in the future (i.e., “your family financial situation in few months”); and their education (i.e., “this situation could negatively affect your studies”). This measure was created considering the main results of previous studies about youth concerns [12,23]. Participants reported their level of concern with each statement on a scale ranging from 1 (nothing) to 5 (a lot). Cronbach’s alpha for this study was 0.73.

Daily routines during the confinement: To assess routines during the confinement, participants were asked to indicate the frequency with which they engaged in a set of five activities on a daily basis. These activities included the following: “maintaining a routine (e.g., getting up, eating… at the same time)”; “doing physical or sports activities”; “doing intellectual activities (e.g., studying and reading)”; “doing leisure activities (e.g., playing games, watching a series, or listening to music)”; and “doing creative activities (e.g., writing and handcrafting)”. A pilot study with nine adolescents was conducted to explore if this pool of items covered all the possible daily routines during the confinement. Participants agreed that no other daily routine should be included, so this measure was tested in the full sample. Items were answered on a frequency scale ranging from 1 (never) to 5 (many times).

Online peer activities during the confinement: Online activities that participants engaged in during the confinement to feel supported, loved, or cared for by their friends were measured with an ad hoc seven-item questionnaire. These activities included: “sharing personal pictures or videos of activities I do at home”; “sharing funny memes or videos”; “messaging on WhatsApp, Telegram, or others”; “making calls or video calls”; “playing online”; “doing challenges”; and “doing activities simultaneously with friends (e.g., watching series, doing homework, and playing sports)”. Items were chosen based on a pilot study with nine participants who were asked about the online activities that they were doing during confinement. Items were answered on a frequency scale ranging from 1 (never) to 5 (many times). Cronbach’s alpha for this study was 0.67.

Feelings of frustration: The general sense of frustration during the confinement was captured using a single item created for this study. Participants were asked “in the past two weeks, to what extent did you feel frustrated?” [35]. Response options were on a Likert scale from 1 (nothing) to 5 (very much).

Optimism: Dispositional optimism, deemed as the general expectation that good things will happen, was measured with the three-item optimism subscale of the Comprehensive Inventory of Thriving [36]. Respondents rated items (e.g., “I have a positive outlook on life”) on a scale ranging from 1 (strongly disagree) to 5 (strongly agree). Cronbach’s alpha for this study was 0.85.

### 2.3. Plan of Analysis

First, to examine adolescents’ concerns about the impact of COVID-19, their daily routines during the COVID-19 lockdown, and the online peer activities used to help youths feel supported during the confinement, descriptive statistics were computed. Besides, three different two-way multivariate analyses of variance (MANOVAs) were conducted to examine mean differences in adolescents’ concerns about COVID-19, their daily routines, and online peer activities based on sex, age groups, and the interaction between sex and age. Second, multiple regression analysis was calculated to examine the association of adolescents’ concerns, their daily routines, online peer activities, and optimism with adolescents’ experiences of frustration. We entered independent variables in the model using the stepwise method. Variables were organized in three blocks: sex and age groups were entered in Block 1; COVID-19-related concerns, online peer activities, and optimism were entered as predictors in Block 2; and finally, the five daily routines were entered in Block 3. The stepwise method used was iterative. The order in which predictors were entered into the model was based on a statistical criterion [37]. Thus, the number of models was not dependent on the number of blocks, but rather on the number of predictors that were significantly associated with the dependent variable. This method began by introducing the independent variable of Block 1 with the highest simple correlation with the outcome. If this predictor significantly improved the percentage of variance explained by the model, it was retained and another predictor was considered. The second predictor included was the next independent variable within the block that had the largest semi-partial correlation with the outcome or, in other words, the predictor that explained the largest part of the remaining variance in the model. If no other variable was identified, it moved on to the next block. The analysis concluded when no more variables from any of the three blocks could make a significant contribution to the predictive power of the model. Each time a variable was introduced, all the statistics of the model were recalculated, resulting in a new model. Collinearity was assessed by calculating tolerance and Variance Inflation Factors (VIF) for each independent variable introduced in the model. The partial eta square (*ηp*^2^) and the coefficient *R*^2^ were used as measures of effect size.

## 3. Results

### 3.1. Concerns about COVID-19 among Youths by Sex and Age

As shown in Table 1, among the top concerns were that a relative could get infected with COVID-19 and that the pandemic could impact studies. In contrast, participants were least concerned about their own health.

Results from MANOVA with the five concerns about COVID-19 as dependent variables and sex and age groups as independent variables revealed significant multivariate effects for both sex, Wilks’ λ = 0.94; *F*(5, 1063) = 14.13, *p* ≤ 0.001, *ηp*^2^ = 0.06, and age, Wilks’ λ = 0.99; *F*(5, 1063) = 2.56, *p* = 0.026, *ηp*^2^ = 0.01. Subsequent univariate ANOVAs on adolescents’ concerns (see Table 1) indicated that girls showed significantly higher levels of concern for all issues than boys. Regarding age differences, the results also indicated that younger adolescents were significantly more worried about their studies and the health of their relatives than their older counterparts. Finally, the multivariate interaction between sex and age was not significant, Wilks’ λ = 0.99; *F*(5, 1063) = 0.77, *p* > 0.05, *ηp*^2^ = 0.00.

### 3.2. Daily Routines of Youths during COVID-19 Confinement by Sex and Age

Regarding routines during the confinement, Table 2 displays descriptive statistics and mean comparisons based on participants’ sex and age. In general, the results indicated that the most frequent activities were intellectual (e.g., studying and reading) and leisure (e.g., playing games, watching a series, and listening to music) activities. In contrast, the least frequent were creative activities (e.g., writing and handcrafting).

Next, a MANOVA, including sex and age as fixed factors and daily routines as dependent variables, yielded significant multivariate effects of sex, Wilks’ λ = 0.92; *F*(5, 1060) = 17.86, *p* ≤ 0.001, *ηp*^2^ = 0.08, and age, Wilks’ λ = 0.99; *F*(5, 1060) = 3.14, *p* = 0.008, *ηp*^2^ = 0.02, on adolescents’ daily routines during COVID-19 confinement. Next, univariate ANOVAs (see Table 2) indicated that girls and late adolescents were more likely to maintain daily routines during the confinement. Girls were more engaged in intellectual, creative, and sports activities on a daily basis. Similarly, younger adolescents reported doing leisure activities with greater frequency than older adolescents. No multivariate interaction effect between sex and age was found, Wilks’ λ = 0.99; *F*(5, 1060) = 1.03, *p* > 0.05, partial *η*^2^ = 0.00.

### 3.3. Online Peer Activities Used to Help Youths Feel Supported by Friends during COVID-19 Confinement by Sex and Age

Table 3 provides an overview of descriptive statistics for online peer activities during the confinement by adolescent sex and age. As shown, participants engaged in online activities to maintain relationships with friends quite regularly. Among the most frequent were activities aimed at maintaining communication (i.e., through messaging on WhatsApp, Telegram, or others, or making calls or video calls) and having companionship (i.e., doing leisure activities simultaneously with friends).

A MANOVA with sex and age as independent variables and online peer activities as dependent variables provided evidence of multivariate effects for both sex, Wilks’ λ = 0.82; *F*(7, 1189) = 36.99, *p* ≤ 0.001, *ηp*^2^ = 0.18, and age, Wilks’ λ = 0.97; *F*(7, 1189) = 5.25, *p* ≤ 0.001, *ηp*^2^ = 0.03. As shown in Table 3, separate univariate ANOVAs on the outcome variables revealed that girls used the internet more extensively than boys to maintain relationships with their friends. While boys played online games with peers more often than girls, girls shared more pictures or videos of themselves doing activities at home, used WhatsApp or Telegram more often to message their friends, did more challenges, and got involved in more simultaneous activities with their friends using the internet. Similarly, when comparing online activities across age groups, the results showed that the younger group of adolescents made more calls or video calls with their friends, were more engaged in challenges, and did more activities simultaneously with their peers. Finally, a multivariate interaction effect between sex and age was observed, Wilks’ λ = 0.98; *F*(7, 1189) = 3.04, *p* = 0.004, *ηp*^2^ = 0.02. Subsequent univariate analyses indicated that the interaction between sex and age was significant for the activities of sharing videos or memes, *F*(1, 1195) = 6.16, *p* = 0.013, *ηp*^2^ = 0.01, making calls or video calls, *F*(1, 1195) = 4.50, *p* = 0.034, *ηp*^2^ = 0.01, and playing online games, *F*(1, 1195) = 7.26, *p* = 0.007, *ηp*^2^ = 0.01; with boys in the youngest age group doing these activities more frequently than boys in the older age group.

### 3.4. Feelings of Frustration during the Confinement: The Role of Adolescents’ Sex, Age, Concerns, Daily Routines, Online Peer Activities, and Optimism

The multiple regression (see Table 4) revealed that sex, optimism, COVID-19-related concerns, online peer activities, maintaining daily routines, and leisure activities contributed significantly to the regression model, *F*(6, 1052) = 31.80, *p* ≤ 0.001. Together the six independent variables accounted for 15.4% of the variance in frustration. Being female, experiencing more concerns about the impacts of COVID-19, and doing online peer activities more frequently were positively related to frustration. In contrast, higher levels of optimism, maintaining daily routines, and doing more leisure activities were negatively associated with frustration.

Regarding collinearity, tolerance values ranged from 0.88 to 0.92 and VIF values ranged from 1.08 to 1.13. These values indicate that the variables introduced in the model were not highly correlated and there was no collinearity among the independent variables.

As a follow up, correlations between adolescents’ frustration and specific aspects of COVID-19-related concerns and online peer activities were computed. In relation to COVID-19-related concerns, frustration was positively associated with concerns about their relatives getting COVID-19 (*r* = 0.08, *p* = 0.010), their family financial strain, currently (*r* = 0.11, *p* ≤ 0.001), and in the future (*r* = 0.13, *p* ≤ 0.001) as well as about their own education (*r* = 0.20, *p* ≤ 0.001). Regarding online peer activities, positive, although small correlations, were found between frustration and sharing personal pictures or videos (*r* = 0.07, *p* = 0.017), messaging friends on WhatsApp, Telegram, or others (*r* = 0.06, *p* = 0.038), and making calls or video calls (*r* = 0.08, *p* = 0.013).

## 4. Discussion

This paper aimed to provide insight into the concerns that young people experienced during the COVID-19 confinement in Spain as well as to explore daily and online activities. Most previous studies have analyzed the psychological impact of COVID-19 in the general or university population, but there are still few studies focused on adolescents. Besides, considering that the progress of the pandemic is still uncertain, this study has also described the degree of frustration experienced by adolescents and tried to identify factors associated with it.

The first aim of this study was to explore adolescents’ concerns about COVID-19. According to the hypotheses of this study, findings evidenced that adolescents were the most worried about the risk of their relatives getting sick and the least worried about their own risk of infection. Previous studies have also shown this result [14]. From a developmental perspective, the lesser degree of concern that adolescents showed about their own health may be explained from the hypothesis of the “personal fable”. Previous studies suggest that a characteristic of adolescent thinking is their propensity to regard themselves as invulnerable. That is, they think that problems and difficulties are not going to happen to them. This cognitive feature is associated with greater involvement in risky behaviors [38]. In times of pandemic, breaking the social distancing guidelines or not following health and safety measures may be considered as new risk behaviors. As such, if adolescents are less concerned about their own health, they may become more involved in irresponsible behaviors that put everyone’s health at risk [39,40]. For this reason, future studies may want to analyze the effectiveness of prevention campaigns aimed at fostering social responsibility. Perhaps personalizing the consequences of adolescents’ risk behaviors on the health of their relatives (e.g., grandparents) may be a more effective way to achieve greater adherence to prevention measures. Beyond health concerns, the findings of this study also indicated that, consistent with research on university students [12], adolescents were very concerned about their studies. A review by Sahu [41] describes that some young people have had to adapt to online education with limited resources at home (e.g., teenagers have had to share computer equipment with different family members who need to telework and study). They have also faced an increase in the amount of schoolwork because of continuous evaluation in addition to uncertainty and pressure of highly monitored final evaluations. All of these factors may have been a source of stress for young people. Thus, these results were in line with the hypothesis of this study and underline that it is also crucial to take care of our education. Additionally, the results have contributed to understanding the role of sex and age on adolescents’ experienced concerns. Regardless of the issue, girls were found to be more worried than boys. For age, although in general there were no differences, younger people were more concerned about the health of others and their studies. These results are in line with previous studies with young people and adults, which have reported increased levels of increased worry, fear, anxiety, and depression among girls [14,17,32]. Moreover, contrary to our expectations, the younger groups of participants were more worried than older adolescents. A previous study among Italian adolescents between 13 to 20 years old [14] has shown that older adolescents were moderately more concerned than younger adolescents. However, the study results are similar to previous studies with university and adult samples, which found higher levels of COVID-19 fear and concerns among younger participants [19,24]. Thus, the results highlight the relevance of understanding that the COVID-19 health crisis is not affecting all people equally, so it is necessary to redouble efforts to the most vulnerable.

The second aim of this study was to provide insights into the activities that young people have been participating in during the confinement on a daily basis and with their peers. Findings suggested that adolescents have spent time on intellectual and leisure activities. In contrast, creative and physical activities were undertaken less frequently. In view of these findings, it is important to implement resources aimed at reducing sedentary behaviors and sports activities should be promoted. In this regard, previous theoretical studies have warned that the possible increase in sedentary behavior together with the excessive time spent on technology may have affected the sleep patterns of children and adolescents, with consequent risk to their development and health [42]. Regarding online activities with peers, this study has also yielded interesting results. Before the pandemic, the analysis of the use of new technologies in peer relations was undertaken in the knowledge that young people tend to alternate face-to-face interactions with online interactions. However, the pandemic situation and the lack of in-person contact has provided a unique opportunity to analyze the contribution of the online world to interpersonal relationships. According to the expectations, the results of this study indicated that young people very often used new technologies to feel supported by their friends. The most frequent online activities were conversations via instant messaging applications and the use of new technologies to do activities simultaneously with peers. In contrast, challenges and online games were less frequently undertaken. Girls used the internet more often than boys, and younger people tried to see their friends more, even if it was through the screen, using challenges, or doing the same activities simultaneously with peers. All these interactions, which are related to spending time together, are traditionally known as companionship or intimacy [43]. In-person, higher levels of companionship are associated with better psychological and relational adjustment [44]. However, the role of these relationships on adolescents’ wellbeing is less known when interactions are 100% online.

The third aim of this study was to examine the factors that related to adolescents’ experiences of frustration during the lockdown. Although frustration itself is not an indicator of mental health, prior research has shown that it is a significant predictor of adolescents’ psychological problems [28]. Findings revealed that optimism, followed by sex, COVID-19-related worries, online peer activities, daily routines, and leisure activities were significantly related to higher levels of frustration. Concerning daily routines, the results showed that keeping daily routines and doing leisure activities were related to lower levels of frustration. Possibly, routines during the confinement have been important to foster a sense of normality, providing structure in the uncertain. In further detail, subsequent analyses indicated that adolescents’ more salient concerns about COVID-19 were associated with higher levels of frustration. Specifically, the findings showed that youths who were more worried about their education, the financial situation of their family, or the health of their relatives also reported more frustration. Another important finding concerns the online activities used to keep youths connected with their peers during the confinement. Although correlations were modest, results indicate that youths who spent more time sharing personal pictures or videos, messaging friends, and making calls or video calls experienced greater levels of frustration. This finding might seem counterintuitive because previous literature has shown that intimacy and companionship with peers are related to better psychological adjustment [44]. However, our results may be far from what is expected because the interaction with peers, due to the confinement, has been restricted to the online context. A previous study found that the balance between online and offline communication matters [45]. For example, among university student couples, closeness increased when online and offline communication was balanced (i.e., couples communicated by both means). In contrast, intimacy and closeness decreased when communication was only online. Thus, these results suggested that the online context was useful for socialization, especially when face-to-face interactions were restricted, but that the internet alone could not compensate or replace face-to-face interaction. Finally, it is important to note that higher levels of optimism were significantly related to lower rates of frustration. This result is consistent with research on the stress buffering effect of optimism [46]. Literature on the psychological impact of COVID-19 has also evidenced that optimism mediates the relationship between COVID-19-related stress and psychological problems, and is associated with lower levels of depression or anxiety, among others [47,48]. Perhaps adolescents with a more optimistic perspective reappraised the situation in a more positive way [49]. Thus, although further studies with adolescents are needed, the results seem to indicate that keeping an optimistic perspective is important in mitigating the psychological impacts of COVID-19.

### Limitations

Despite the strengths of this study, some limitations should be outlined. First, the sample was mostly composed of young people from the southern area of Spain. Although the aim of the study was not to make comparisons between different areas of the country, future studies could benefit from a stratified sampling. Second, adolescents’ frustration was assessed through a single-item questionnaire. The main reason was that online surveys should be short to facilitate participation. However, future studies could evaluate this construct through a validated multi-item instrument. Third, the cross-sectional design of this study did not allow for an analysis of the directionality of the relationships between the variables. Future longitudinal studies could explore the temporal association between the factors associated with adolescents’ frustration. Finally, given that data were collected during the strict confinement period in Spain, this study assumed that participants were not having any actual face-to-face contact with their peers and that all their social routines were online. However, as they were not explicitly asked about the extent to which they followed the strict isolation measures imposed, it remains possible that a few individuals in the study had had actual contact with their friends.

## 5. Conclusions

The pandemic has led to moderate-to-high levels of frustration among adolescents. The results of this study have allowed us to delve deeper into the role that concerns, daily activities, online interactions with friends, and optimism play in the degree of frustration that young people have experienced. First, matters related to family health, family financial situation, and education were found to be associated with more frustration. Among these, the academic concern was the most relevant variable because of the magnitude of the association with adolescents’ frustration. In this sense, it is necessary to develop an educational response that involves the different stakeholders (politicians, teachers, families, and students) to ensure that we take care of both students’ physical and mental health. To this end, it is necessary to rethink the changes that should be implemented in order to prepare schools for a new COVID-19 outbreak. Second, findings have allowed us to make conclusions about the importance of adolescents maintaining a daily routine, and participating in physical, leisure, and creative activities. Special attention should be paid to creative activities because, despite being negatively related to frustration, they were the least frequent activity undertaken during confinement. Third, another relevant conclusion of this study was that optimism turned out to be the variable that showed a stronger negative association with adolescents’ frustration. Thus, it seems that being able to reinterpret the situation positively represents a precious resource for adolescents in facing this health crisis. In summary, this work contributes to understanding the emotional impact that the COVID-19 crisis has on Spanish adolescents, exploring not only the factors that are related to more significant psychological effects but also to some variables that are associated with greater resilience.

## Figures and Tables

**Table 1:** Descriptive and MANOVA statistics for adolescents’ concerns by sex and age groups.

Concerns about COVID-19	Total	Sex				Age Groups			
		Boys	Girls			16–18 Years	19–25 Years		
	*M* (*SD*)	*M* (*SD*)	*M* (*SD*)	*F* (*1, 1067*)	*ηp* ^2^	*M* (*SD*)	*M* (*SD*)	*F* (*1, 1067*)	*ηp* ^2^
Their own health	2.84 (1.25)	2.54 (1.23)	2.95 (1.24)	22.98 ***	0.02	2.89 (1.24)	2.80 (1.26)	2.06	0.00
Others health	4.29 (0.98)	4.04 (1.11)	4.39 (0.91)	29.41 ***	0.03	4.38 (0.92)	4.22 (1.02)	4.74 *	0.00
Current family financial situation	3.27 (1.35)	3.00 (1.32)	3.37 (1.35)	18.46 ***	0.03	3.28 (1.35)	3.25 (1.35)	0.00	0.00
Future family financial situation	3.55 (1.35)	3.25 (1.37)	3.66 (1.33)	20.31 ***	0.02	3.60 (1.33)	3.51 (1.37)	0.73	0.00
Their own education	4.25 (1.12)	3.89 (1.30)	4.40 (1.01)	47.49 ***	0.04	4.36 (1.02)	4.17 (1.19)	7.05 **	0.01

* *p* < 0.05, ** *p* < 0.01, *** *p* ≤ 0.001. Abbreviations: MANOVA, Multivariate Analysis of Variance; *M*, Mean; *F*, F-Snedecor; *SD*, Standard Deviation; *ηp*^2^, partial eta square.

**Table 2:** Descriptive and MANOVA statistics for daily routines by sex and age groups.

Daily Routines	Total	Sex				Age Groups			
		Boys	Girls			16–18 Years	19–25 Years		
	*M* (*SD*)	*M* (*SD*)	*M* (*SD*)	*F* (*1, 1064*)	*ηp* ^2^	*M* (*SD*)	*M* (*SD*)	*F* (*1, 1064*)	*ηp* ^2^
Maintaining a routine	3.49 (1.25)	3.21 (1.25)	3.61 (1.24)	23.20 ***	0.02	3.38 (1.25)	3.58 (1.25)	8.03 **	0.01
Physical or sports activities	3.31 (1.32)	3.18 (1.37)	3.36 (1.29)	4.51 *	0.00	3.30 (1.29)	3.31 (1.33)	0.18	0.00
Intellectual activities	4.18 (1.00)	3.82 (1.14)	4.33 (0.90)	58.79 ***	0.05	4.22 (0.99)	4.16 (1.01)	0.02	0.00
Leisure activities	4.26 (0.93)	4.33 (0.91)	4.24 (0.93)	2.38	0.00	4.36 (0.90)	4.19 (0.94)	6.85 **	0.01
Creative activities	2.59 (1.34)	2.24 (1.36)	2.72 (1.31)	29.77 ***	0.03	2.61 (1.34)	2.56 (1.35)	0.01	0.00

* *p* < 0.05, ** *p* < 0.01, *** *p* ≤ 0.001. Abbreviations: MANOVA, Multivariate Analysis of Variance; *M*, Mean; *F*, F-Snedecor; *SD*, Standard Deviation; *ηp*^2^, partial eta square.

**Table 3:** Descriptive and MANOVA statistics for online peer activities by sex and age groups.

Online Peer Activities	Total	Sex				Age Groups			
		Boys	Girls			16–18 Years	19–25 Years		
	*M* (*SD*)	*M* (*SD*)	*M* (*SD*)	*F* (*1, 1195*)	*ηp* ^2^	*M* (*SD*)	*M* (*SD*)	*F* (*1, 1195*)	*ηp* ^2^
Sharing personal pictures or videos	3.14 (1.25)	2.72 (1.20)	3.31 (1.22)	60.92 ***	0.05	3.14 (1.26)	3.14 (1.23)	0.78	0.00
Sharing memes or funny videos	3.71 (1.23)	3.73 (1.24)	3.70 (1.23)	0.34	0.00	3.77 (1.24)	3.66 (1.22)	6.06 *	0.00
Messaging: WhatsApp, Telegram	4.57 (0.79)	4.36 (0.93)	4.66 (0.70)	31.10 ***	0.03	4.60 (0.77)	4.55 (0.80)	2.81	0.00
Making calls or video calls	3.88 (1.05)	3.80 (1.08)	3.91 (1.04)	3.08	0.00	3.98 (1.03)	3.81 (1.06)	13.04 ***	0.01
Playing online	2.88 (1.46)	3.52 (1.44)	2.62 (1.40)	108.02 ***	0.08	2.97 (1.46)	2.82 (1.46)	6.46 *	0.01
Doing challenges	2.05 (1.09)	1.84 (1.00)	2.14 (1.11)	21.94 ***	0.02	2.21 (1.11)	1.94 (1.06)	15.41 ***	0.01
Doing activities simultaneously with friends	4.12 (1.20)	3.88 (1.25)	4.22 (1.17)	21.21 ***	0.02	4.31 (1.04)	3.97 (1.29)	13.52 ***	0.01

* *p* < 0.05, *** *p* ≤ 0.001. Abbreviations: MANOVA, Multivariate Analysis of Variance; *M*, Mean; *F*, F-Snedecor; *SD*, Standard Deviation; *ηp*^2^, partial eta square.

**Table 4:** Results of the multiple linear regression on adolescents’ frustration.

Model	Variables	*B*	*t*	Δ*R*^2^
1	Sex	0.34	4.75 ***	0.02
2	Sex	0.30	4.50 ***	0.12
	Optimism	−0.32	−10.79 ***	
3	Sex	0.23	3.41 **	0.14
	Optimism	−0.32	−10.94 ***	
	COVID−19-related concerns	0.17	4.70 ***	
4	Sex	0.23	3.36 **	0.14
	Optimism	−0.33	−11.15 ***	
	COVID−19-related concerns	0.16	4.23 ***	
	Online peer activities	0.10	2.10 *	
5	Sex	0.27	3.87 ***	0.15
	Optimism	−0.31	−10.09 ***	
	COVID−19-related concerns	0.15	4.20 ***	
	Online peer activities	0.09	1.98 *	
	Maintaining daily routines	−0.08	−3.28 **	
6	Sex	0.26	3.82 ***	0.15
	Optimism	−0.30	−9.91 ***	
	COVID−19-related concerns	0.14	3.87 ***	
	Online peer activities	0.12	2.54 *	
	Maintaining daily routines	−0.09	−3.49 **	
	Leisure activities	−0.08	−2.50 *	

*N* = 1059; * *p* < 0.05, ** *p* < 0.01, *** *p* ≤ 0.001. Abbreviations: *B*, Unstandardized beta coefficient; *t*, t-value; Δ*R*^2^, increment in the fraction of the variation in frustration accounted for the independent variables of each model.

## Data Availability

The datasets generated and analyzed during the current study are available from the corresponding author on reasonable request.

## References

[1] Mantovani A., Rinaldi E., Zusi C., Beatrice G., Saccomani M.D., Dalbeni A. (2020). Coronavirus disease 2019 (COVID-19) in children and/or adolescents: A meta-analysis. Pediatric Res..

[2] UNESCO COVID-19 Educational Disruption and Response. https://en.unesco.org/covid19/educationresponse.

[3] Lee J. (2020). Mental health effects of school closures during COVID-19. Lancet Child Adolesc. Health.

[4] Larson R.W., Eccles J.S. (2005). Organized Activities as Contexts of Development: Extracurricular Activities, after-School and Community Programs.

[5] DPhil A., Tomova L., Blakemore S.J. (2020). The effects of social deprivation on adolescent development and mental health. Lancet Child Adolesc. Health.

[6] Luchetti M., Lee J.H., Aschwanden D., Sesker A., Strickhouser J.E., Terracciano A., Sutin A.R. (2020). The trajectory of loneliness in response to COVID-19. Am. Psychol..

[7] Prime H., Wade M., Browne D.T. (2020). Risk and resilience in family during the COVID-19 pandemic. Am. Psychol..

[8] Fergert J., Vitielli B., Plener P., Clemens V. (2020). Challenges and burden of the Coronavirus 2019 (COVID-19) pandemic for child and adolescent mental health: A narrative review to highlight clinical and research needs in the acute phase and the long return to normality. Child Adolesc. Psychiatry Ment. Health.

[9] Wang G., Zhang Y., Zhao J., Zhang J., Jiang J. (2020). Mitigate the effects of home confinement on children during the COVID-19 outbreak. Lancet.

[10] Liang L., Ren H., Cao R., Hu Y., Qin Z., Li C., Mei S. (2020). The effect of COVID-19 on youth mental health. Psychiatr. Q..

[11] Romero E., López-Romero L., Domínguez-Álvarez B., Villar P., Gómez-Fraguela J.A. (2020). Testing the effects of COVID-19 confinement in Spanish children: The role of parents’ distress, emotional problems and specific parenting. Int. J. Environ. Res. Public Health.

[12] Wang C., Zhao H. (2020). The impact of COVID-19 on anxiety in Chinese university students. Front. Psychol..

[13] Yang H., Bin P., Jingwei A. (2020). Opinions from the epicenter: An online survey of university students in Wuhan amidst the COVID-19 outbreak. J. Chin. Gov..

[14] Buzzi C., Tucci M., Ciprandi R., Brambilla I., Caimmi S., Ciprandi G., Marseglia G.L. (2020). The psycho-social effects of COVID-19 on Italian adolescents’ attitudes and behaviors. Ital. J. Pediatr..

[15] Alvis L., Shook N., Oosterhoff B. Adolescents’ Prosocial Experiences During the COVID-19 Pandemic: Associations with Mental Health and Community Attachments. PsyArXiv.

[16] Gao J., Zheng P., Jia Y., Chen H., Mao Y., Chen S., Wang Y., Fu H., Dai J. (2020). Mental health problems and social media exposure during COVID-19 outbreak. PLoS ONE.

[17] Gritsenko V., Skugarevsky O., Konstantinov V., Khamenka N., Marinova T., Reznik A., Isralowitz R. (2020). COVID 19 fear, stress, anxiety, and substance use among Russian and Belarusian university students. Int. J. Ment. Health Addict..

[18] Pérez-Fuentes M.C., Molero M.M., Martos A., Gázquez Linares J.J. (2020). Threat of COVID-19 and emotional state during quarantine: Positive and negative affect as mediators in a cross-sectional study of the Spanish population. PLoS ONE.

[19] Reznik A., Gritsenko V., Konstantinov V., Khamenka N., Isralowitz R. (2020). COVID-19 Fear in Eastern Europe: Validation of the Fear of COVID-19 Scale. Int. J. Ment. Health Addict..

[20] Seçer I., Ulaş S. (2020). An investigation of the effect of COVID-19 on OCD in youth in the context of emotional reactivity, experiential avoidance, depression and anxiety. Int. J. Ment. Health Addict..

[21] Liu N., Zhang F., Wei C., Jia Y., Shang Z., Sun L., Wu L., Sun Z., Zhou Y., Wang Y. (2020). Prevalence and predictors of PTSS during COVID-19 outbreak in China hardest-hit areas: Gender differences matter. Psychiatry Res..

[22] Baloran E.T. (2020). Knowledge, attitudes, anxiety, and coping strategies of students during COVID-19 pandemic. J. Loss Trauma.

[23] Odriozola-González P., Planchuelo-Gómez Á., Irurtia M.J., de Luis-García R. (2020). Psychological effects of the COVID-19 outbreak and lockdown among students and workers of a Spanish university. Psychiatry Res..

[24] Sorokin M.Y., Kasyanov E.D., Rukavishnikov G.V., Makarevich O.V., Neznanov N.G., Lutova N.B., Mazo G.E. (2020). Structure of anxiety associated with COVID-19 pandemic: The online survey results. Bull. RSMU.

[25] Cohen A., Hoyt L., Dull B. (2020). A Descriptive study of coronavirus disease 2019-related experiences and perspectives of a national sample of college students in spring 2020. J. Adolesc. Health.

[26] Brooks S.K., Webster R.K., Smith L.E., Woodland L., Wessely S., Greenberg N., Rubin G.J. (2020). The psychological impact of quarantine and how to reduce it: Rapid review of the evidence. Lancet.

[27] Ryan R.M., Deci E.L., Vansteenkiste M., Cichetti D. (2015). Autonomy and autonomy disturbances in self-development and psychopathology: Research on motivation, attachment, and clinical process. Developmental Psychopathology.

[28] Vansteenkiste M., Ryan R.M. (2013). On psychological growth and vulnerability: Basic psychological need satisfaction and need frustration as a unifying principle. J. Psychother. Integr..

[29] Dvorsky M., Breaux R., Becker S.P. (2020). Finding ordinary magic in extraordinary times: Child and adolescent resilience during the COVID-19 pandemic. Eur. Child Adolesc. Psychiatry.

[30] World Health Organization Mental Health and Psychosocial Considerations during the COVID-19 Outbreak, 18 March 2020. https://apps.who.int/iris/bitstream/handle/10665/331490/WHO-2019-nCoV-MentalHealth-2020.1-eng.pdf.

[31] Brown B.B., Larson J., Lerner R.M., Steinberg L. (2009). Peer relationships in adolescence. Handbook of Adolescent Psychology: Contextual Influences on Adolescent Development.

[32] Zhou S.-J., Zhang L.-G., Wang L.-L., Guo Z.-C., Wang J.-Q., Chen J.-C., Liu M., Chen C., Chen J.-X. (2020). Prevalence and socio demographic correlates of psychological health problems in Chinese adolescents during the outbreak of COVID 19. Eur. Child Adolesc. Psychiatry.

[33] Xie X., Xue Q., Zhou Y., Zhu K., Liu Q., Zhang J., Song R. (2020). Mental health status among children in home confinement during the coronavirus disease 2019 outbreak in Hubei Province, China. JAMA Pediatr..

[34] Steinberg L. (2014). Age of Opportunity: Lessons from the New Science of Adolescence.

[35] Ezpeleta L., Navarro J., de la Osa N., Trepat E., Penelo E. (2020). Life conditions during COVID-19 lockdown and mental health in Spanish adolescents. Int. J. Environ. Res..

[36] Su R., Tay L., Diener E. (2014). The development and validation of the Comprehensive Inventory of Thriving (CIT) and the Brief Inventory of Thriving (BIT). Appl. Psychol. Health Well Being.

[37] Field A. (2014). Regression. Discovering Statistics using IBM SPSS Statistics.

[38] Alberts A., Elkind D., Ginsberg S. (2007). The personal fable and risk-taking in early adolescence. J. Youth Adolesc..

[39] Abbott A., Askelson N., Scherer A.M., Afifi R.A. (2020). Critical reflections on COVID-19 communication efforts targeting adolescents and young adults. J. Adolesc. Health.

[40] Oosterhoff B., Palmer C. Psychological Correlates of News Monitoring, Social Distancing, Disinfecting, and Hoarding Behaviors among US Adolescents during the COVID-19 Pandemic. PsyArXiv.

[41] Sahu P. (2020). Closure of universities due to Coronavirus disease 2019 (COVID-19): Impact on education and mental health of students and academic staff. Cureus.

[42] Becker S.P., Gregory A.M. (2020). Editorial Perspective: Perils and promise for child and adolescent sleep and associated psychopathology during the COVID-19 pandemic. J. Child Psychol. Psychiatry.

[43] Furman W., Buhrmester D. (2009). The Network of Relationships Inventory: Behavioral systems version. Int. J. Behav. Dev..

[44] Zimmer-Gembeck M.J. (2002). The development of romantic relationships and adaptations in the system of peer relationships. J. Adolesc. Health.

[45] Caughlin J.P., Sharabi L.L. (2013). A communicative interdependence perspective of close relationships: The connections between mediated and unmediated interactions matter. J. Commun..

[46] Lai J. (2009). Dispositional optimism buffers the impact of daily hassles on mental health in Chinese adolescents. Personal. Individ. Differ..

[47] Arslan G., Yildirim M. Coronavirus stress, meaningful living, optimism, and depressive symptoms: A study of moderated mediation model. PsyArXiv.

[48] Arslan G., Yildirim M., Tanhan A., Buluş M., Allen K. (2020). Coronavirus stress, optimism-pessimism, psychological inflexibility, and psychological health: Psychometric properties of the coronavirus stress measure. Int. J. Ment. Health Addict..

[49] Calvete E., Connor-Smith J.K. (2006). Perceived social support, coping, and symptoms of distress in American and Spanish students. Anxiety Stress Coping.

